# Histone deacetylase 5 regulates the inflammatory response of macrophages

**DOI:** 10.1111/jcmm.12595

**Published:** 2015-06-08

**Authors:** Lukas Poralla, Thorsten Stroh, Ulrike Erben, Marie Sittig, Sven Liebig, Britta Siegmund, Rainer Glauben

**Affiliations:** Medical Department I (Gastroenterology, Rheumatology, Infectious Diseases), Charité – Universitätsmedizin BerlinBerlin, Germany

**Keywords:** histone deacetylase, macrophage, inflammation, cytokine, tumour necrosis factor

## Abstract

Modifying the chromatin structure and interacting with non-histone proteins, histone deacetylases (HDAC) are involved in vital cellular processes at different levels. We here specifically investigated the direct effects of HDAC5 in macrophage activation in response to bacterial or cytokine stimuli. Using murine and human macrophage cell lines, we studied the expression profile and the immunological function of HDAC5 at transcription and protein level in over-expression as well as RNA interference experiments. Toll-like receptor-mediated stimulation of murine RAW264.7 cells significantly reduced HDAC5 mRNA within 7 hrs but presented baseline levels after 24 hrs, a mechanism that was also found for Interferon-γ treatment. If treated with lipopolysaccharide, RAW264.7 cells transfected for over-expression only of full-length but not of mutant HDAC5, significantly elevated secretion of tumour necrosis factor α and of the monocyte chemotactic protein-1. These effects were accompanied by increased nuclear factor-κB activity. Accordingly, knock down of HDAC5-mRNA expression using specific siRNA significantly reduced the production of these cytokines in RAW264.7 or human U937 cells. Taken together, our results suggest a strong regulatory function of HDAC5 in the pro-inflammatory response of macrophages.

## Introduction

Gene transcription is regulated by multiple mechanisms including modifications of the DNA itself or of changes in DNA-associated histones. Histone acetyltransferases mediate acetylation of the ε-amino groups of lysine and histone deacetylases (HDAC) remove these acetyl groups. The eponymous function of the latter resulting in chromatin compaction inhibits transcription factor binding 1. HDAC also modify non-histone proteins often involved in crucial functions like DNA repair and replication, metabolism or cellular signalling like p53, hypoxia-inducible factor 1α, STAT3 or p65 2.

HDAC are evolutionarily highly conserved proteins. HDAC5 belongs to class II of the classical family sharing a zinc-dependent catalytic domain 3. Regulated by phosphorylation of serine residues at the N-terminus of the enzyme, class II HDAC are able to shuttle between nucleus and cytoplasm 4,5. This capacity to translocate enables the interaction with cytoplasmic non-histone proteins. The enzymatic activity is effectively blocked by HDAC inhibitors, representing excellent tools to evaluate HDAC function *in vitro* or *in vivo*. Originally approved as anti-cancer agents 6,7, low dose HDAC inhibitors were proven also anti-inflammatory, leading to several clinical trials *e.g*. for systemic-onset juvenile idiopathic arthritis 8. This concept is strongly supported by experimental data indicating an anti-inflammatory effect for HDAC inhibitors in murine models of chronic inflammation such as rheumatoid arthritis 9, hepatitis 10 and various models of colitis 11 or inflammation-associated cancer 12. At cellular level, CD4^+^ T cells, dendritic cells and/or macrophages are shown to be major players 13.

Macrophages connect innate and adaptive immune responses by phagocytosis of extracellular pathogens and antigen presentation *via* MHC II. While anti-inflammatory macrophages prevent overwhelming immune responses by secretion of IL-10, pro-inflammatory macrophages enhance the immune response by secretion of pro-inflammatory cytokines such as tumour necrosis factor (TNF)α or monocyte chemotactic protein (MCP)-1 14,15. Thus, macrophages are critical in immune homeostasis and macrophage dysfunction strongly associates to the pathogenesis of chronic inflammatory conditions including inflammatory bowel diseases (IBD) 16. Suggesting that HDAC in macrophages in general rather convey a pro-inflammatory function, secretion of pro-inflammatory cytokines such as TNFα, IL-1α, IL-1β and IFNγ by human mononuclear cells and murine bone marrow-derived macrophages was suppressed in the presence of HDAC inhibitors 17,18.

As for the contribution of single HDAC in complex processes, increased expression of HDAC5 in human alveolar epithelial cells after infection with *Legionella pneumophila* indicates a central role fort HDAC5 in host defense 19. Our group revealed a pro-inflammatory function of HDAC5 by a specific knock-down in CD4^+^ T cells (unpublished data). For HDAC3 in macrophages in general and in IBD regarding the intestinal epithelial cells in particular, an important role in the pro-inflammatory response could be demonstrated 20,21. These studies prove, that single HDAC are able to act upon activation in adult (immune) cells, especially in macrophages, and are not restricted to developmental or cancer biology.

Addressing the potential role of HDAC5 in chronic inflammation, we here specifically asked whether HDAC5 expression and function directly affect the response of macrophages to pro- or anti-inflammatory stimuli. Making use of murine and human macrophage cell lines, we studied the effect of HDAC5 over-expression and knock-down on nuclear factor kappa B (NF-κB)-dependent regulation of cytokine and chemokine expression.

## Materials and methods

### Cell lines

The murine macrophage cell line RAW264.7 (ATCC TIB71; American Type Culture Collection, Manassas, VA) and the human histiocytic lymphoma cell line U937 (ACC5; DSMZ – German Collection of Microorganisms and Cell Cultures*,* Braunschweig, Germany) were cultured according to the provider’s instructions and routinely checked for mycoplasma contamination by mycoplasm-specific PCR (e-Myco™ plus; Intron Biotechnology, Sangdaewon-Dong, South Korea).

### *In vitro* stimulation of cells

U937 cells were differentiated into macrophage-like cells by 10 ng/ml phorbol 12-myristate 13-acetate (Sigma-Aldrich, St. Louis, MO, USA) within 24 hrs before stimulation with 10 ng/ml lipopolysaccharide(LPS; *Escherichia coli* 011:B4; Invivogen, San Diego, CA, USA). RAW264.7 cells were stimulated with 1 μg/ml LPS, 5 ng/ml TNFα (Peprotech, New Jersey, NJ, USA), 20 ng/ml IL-4 (R&D Systems, Minneapolis, MN, USA), 20 ng/ml IL-13 (Peprotech), 20 ng/ml macrophage colony-stimulating factor (M-CSF; Peprotech), 20 ng/ml granulocyte-macrophage colony-stimulating factor (GM-CSF; Peprotech), 20 ng/ml MCP-1 (Peprotech), 20 ng/ml IFNγ (Peprotech), 500 nM CpG oligodeoxynucleotide (ODN 2088, 5′-CCTGGCGGGGAAGT-3′; TIB Molbiol, Berlin, Germany), 20 ng/ml IL-4 and 20 ng/ml IL-6 (Peprotech) or 5 ng/ml TGFβ (Miltenyi Biotech, Bergisch-Gladbach, Germany). Cell culture supernatants and cells were collected separately, frozen over liquid nitrogen and stored at −80°C.

### Transfection of cells

Lipopolysaccharide-free plasmid DNA (6 μg) and siRNA (120 pmol; Qiagen, Hilden, Germany) was transferred by electroporation as described previously 22. RAW264.7 cells received 600 V for 100 μsec. followed by 100 V for 10 msec.; U937 cells 400 V for 400 μsec. 22. For HDAC5 over-expression RAW264.7 cells were transfected with a pCMX-based vector encoding for HA-tagged mouse HDAC5 (pHDAC5; 8267 bp) kindly provided by Ronald Evans (Salk Institute, San Diego, CA, USA) 23. To determine NF-κB activation, RAW264.7 cells were transfected with an NF-κB reporter plasmid containing a single copy of an NF-κB response element linked to a luciferase sequence (pGL4.10; 4242 bp; Clontech, Mountain View, CA, USA). In RAW264.7 cells HDAC5 expression was knocked down by transfecting HDAC5-specific (siHDAC5) or control siRNA (siCtrl; Table1).

**Table 1:** Oligonucleotides used for knock down, mRNA quantification and *in vitro* mutagenesis

Purpose	Target/name	Dir.	Sequence (5′–3′)
siRNA*	MmHDAC5_2	S	GCCUCGGAACCCAACUUAATT
		AS	UUAAGUUGGGUUCCGAGGCCG
	MmHDAC5_5	S	GGGCAAGAUCCUUACCAAATT
		AS	UUUGGUAAGGAUCUUGCCCAG
	HsHDAC5_1	S	ACGACACGUUCAUGCUAAATT
		AS	UUUAGCAUGAACGUGUCGUAG
	HsHDAC5_4	S	CGGGUUUGAUGCUGUUGAATT
		AS	UUCAACAGCAUCAAACCCGGC
	Control	S	UUCUCCGAACGUGUCACGUdTdT
		AS	ACGUGACAGGUUCGGAGAAdTdT
mRNA quantification	MmHDAC4	S	ACTGCCCTTGGAACCTGCATT
		AS	ATGCAACTGTGCCTCATGCTGT
	MmHDAC5	S	ACGTTCATGCTGAAGCACCAGT
		AS	ACTTGCCGAGCAGACCAGTTT
	MmHDAC6	S	ACCGTGAAGGTGCCAACT TTGA
		AS	AAAGCAGAAACCACAGGCAGCA
	MmHDAC7	S	TGTCACCGACCTTGCCTTCAAA
		AS	ATCTTGCTGGCTTTGCCGTGTT
	MmHDAC9	S	AAACTGCCTCAGAGCCCAACTT
		AS	AGCAGGGCCATTGTTTGGTGAA
	MmHDAC10	S	AAGGTGCCTGTGTTTGTCAGCTTG
		AS	AGTGAAAGGTGTCCGGGTGAAAGT
	MmGAPDH	S	CATCCTGCACCACCAACTGC
		AS	ACGCCACAGCTTTCCAAGG
	HsHDAC5	S	TCTCGGCTCTGCTCAGTGTA
		AS	CTGCACACAGCTCCAGTGTT
	HsGAPDH	S	GAGCTGAACGGGAAGCTCAC
		AS	GCCTGCTTCACCACCTTCTT
*In vitro* mutagenesis	MmHDAC5 ΔA in ATG	S	CTAGGTACCTCCACCTGAACTCTCCCAACG
		AS	CGTTGGGAGAGTTCAGGTGGAGGTACCTAG
	MmHDAC5 H885-> F885	S	TCTACATCTCCCTGTTTCGCTACGACAACG
		AS	CGTTGTCGTAGCGAAACAGGGAGATGTAGA

* siRNA was purchased from Qiagen; all other primers were from TIB MolBiol.

Mm: murine; Dir: direction; Hs: human; S: sense; AS: antisense.

### Quantification of mRNA expression

RNA (500 ng) prepared from defined cell numbers (Qiagen) was reversely transcribed (Life Technologies, Carlsbad, CA, USA). Complementary DNA was subjected to quantitative PCR (qPCR) using SYBR-Green (Life Technologies) on a StepOne Plus Realtime PCR System (Applied Biosystems, Darmstadt, Germany) using primer pairs specific for murine or human HDAC5 and GAPDH mRNA (Table1). HDAC5 expression was determined in relation to GAPDH as housekeeping gene.

### Quantification of secreted cytokines and chemokines

Supernatants of RAW264.7 and U937 cells were analysed for TNFα, IL-6 or IL-10 by ELISA; MCP-1 of RAW264.7 by cytometric bead array (all BD Bioscience, Heidelberg, Germany) according to the manufacturer’s instructions.

### Western-blot analysis

RAW264.7 or U937 cells were lysed in 20 μl 50 mM Tris-HCl (pH 8.0) containing 20% SDS and 5% β-mercaptoethanol. Murine and human HDAC5 were detected by a rabbit-derived polyclonal antibody (#2082; Cell Signaling Technology, Danvers, MA, USA) and a rabbit-specific secondary antibody (DAKO, Hamburg, Germany). The HA-tag was assessed using a murine monoclonal antibody (clone 6E2; Cell Signaling Technology) and a mouse-specific secondary antibody (DAKO). β-actin served as control for equal protein loading and was detected by a murine monoclonal antibody (AC15; Sigma-Aldrich) and a mouse-specific second antibody (DAKO). Secondary antibody binding was identified by chemiluminescence and protein sizes were approximated relative to a pre-stained molecular weight marker (Thermo Fisher Scientific, Waltham, MA, USA). Densitometric analysis was performed using ImageJ software (NIH, Bethesda, MD, USA).

### *In vitro* mutagenesis

Single base pairs of murine HDAC5 were exchanged by oligonucleotide-directed mutagenesis using plasmid DNA as a template as described earlier 24. Sequence-specific primers (Table1) were designed to delete the adenosine in the start codon (ΔA in ATG; pHDAC5-FS) or to mutate the histidine residue at position 885 to phenylalanine (H885->F885; pHDAC5-F885) 4. Mutated sequences were confirmed by DNA sequencing from the expression plasmid (Seqlab, Göttingen, Germany).

### Luciferase reporter assay

RAW264.7 transfected with pGL4.10 were used with the Luciferase Reporter Gene Detection Kit (Sigma-Aldrich, Munich, Germany) according to the manufacturer’s instructions. Light emission measured in relative light units was assessed for 2 sec. in relation to a luciferase standard curve using a luminometer (Berthold Technologies, Bad Wildbad, Germany).

### Statistical analysis

Statistical significance was determined by Kruskal–Wallis test to compare multiple groups or Mann–Whitney test to compare two groups using GraphPad Prism 5 software for Windows (GraphPad Software, San Diego, CA, USA).

## Results

### Expression of HDAC5 in macrophages

**Figure 1:**
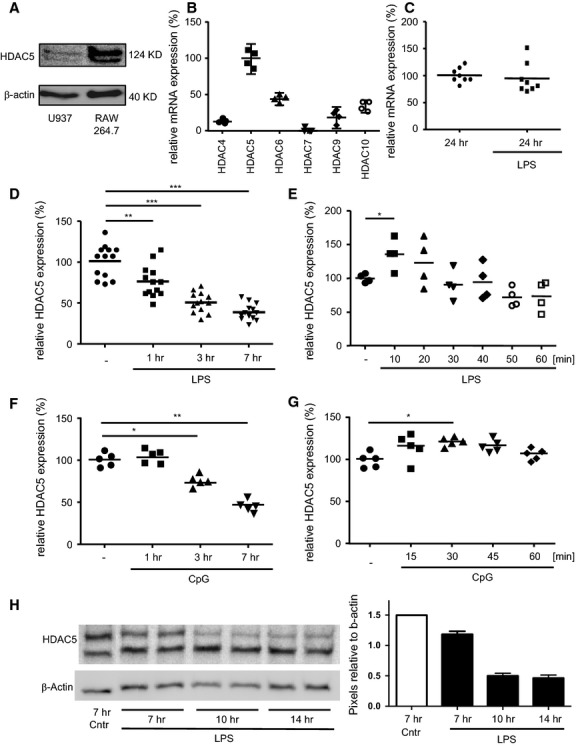
Expression levels of HDAC5 in murine and human macrophages. (A) Lysates from untreated RAW264.7 (10^5^) or U937 (6.6 × 10^4^) cells were analysed by Western blot for HDAC5 and β-actin as loading control. Approximate protein sizes are indicated. Representative image of 4 (RAW264.7) or 2 (U937) independent experiments. (B) RAW264.7 cells were left untreated and mRNA levels of class II HDAC were determined by qPCR relative to HDAC5. *n* = 4 from two independent experiments. (C-E) RAW264.7 cells were left untreated or were stimulated with LPS or CpG before expression levels of HDAC5 mRNA were determined by qPCR relative to the untreated samples. (C) Endpoint after 24 hrs LPS treatment. *n* = 8 from two independent experiments. (D) Kinetics between 0 and 7 hrs LPS treatment. *n* = 13 from six independent experiments. (E) Kinetics between 0 and 60 min. LPS treatment. *n* = 4 from two independent experiments. (F) Kinetics between 0 and 7 hrs CpG treatment *n* = 5 from three independent experiments. (G) Kinetics between 0 and 60 min. CpG treatment *n* = 6 from three independent experiments. (H) Kinetics between 7 and 14 hrs LPS treatment, 10^5^ cells per slot, *y*-axis shows HDAC band intensity relative to the corresponding b-actin band, *n* = 2. Median values and ranges. *P < 0.05, **P < 0.01, ***P < 0.001 by Kruskal–Wallis test and Mann–Whitney test as post test.

The presence of HDAC5 built the prerequisite for all experiments and so we assessed the baseline expression of the enzyme in murine RAW264.7 and human U937 cells. The HDAC5-specific Western blot analyses showed bands of about 130 kD in both cell lines (Fig.1A). As confirmed in the knock down experiments (see below, Fig.4) the upper band represents HDAC5, the lower band is an unspecific staining of the polyclonal anti-HDAC5 antibody. This was confirmed at mRNA level for the RAW264.7 cells (Fig.1B). As the question was whether pro-inflammatory activation influenced HDAC5 expression levels, RAW264.7 cells were also stimulated with LPS. After 24 hrs, the HDAC5 mRNA expression was comparable to the baseline (Fig.1C). Kinetics over 7 hrs revealed a significant decrease of HDAC5 expression by about 70% (Fig.1D), which appeared to revert to the level of non-activated cells after 24 hrs (Fig.1C). Looking at the short-term kinetics within the first 60 min. after activation, a very early significant up-regulation of the HDAC5 mRNA expression in response to LPS is demonstrated (Fig.1E). Corresponding results could be observed using CpG as a toll-like receptor-(TLR) 9 agonist (Fig.1F and G). The down regulation after LPS stimulation after 7, 10 and 14 hrs was confirmed on a protein level *via* western blot (Fig.1H).

**Figure 2:**
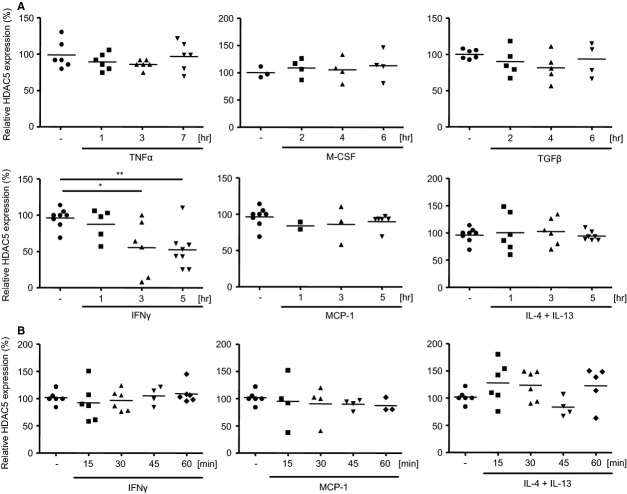
Kinetics of HDAC5 mRNA expression in RAW264.7 cells after cytokine stimulation. (A) Cells (10^6^) were incubated either in absence or presence of TNFα, M-CSF, TGFβ, IFNγ, MCP-1 or IL-4 + IL-13 for up to 7 hrs as indicated. (B) RAW264.7 cells were stimulated with IFNγ, MCP-1 or IL-4 + IL-13 for up to 60 min. as indicated. Expression levels of HDAC5 mRNA were determined by qPCR relative to the untreated samples. Median values and ranges of *n* = 2–8 from 2–4 independent experiments. *P < 0.05, **P < 0.01 Kruskal–Wallis test and Mann–Whitney test as post-test.

Since TNFα belongs to the genes up-regulated early on after LPS stimulation, first the pattern of HDAC5 mRNA expression after stimulation with TNFα was investigated. As shown in Figure2, the presence of TNFα did not affect HDAC5 mRNA expression over the 7-hr time-period investigated. To assess the milieu dependent expression of HDAC5 mRNA, RAW264.7 cells were subjected to defined chemotaxis-inducing, anti-inflammatory, polarizing or pro-inflammatory conditions for 5 to 7 hrs. While the presence of TNFα, M-CSF, TGFβ, MCP-1 or a combination of IL-4 with IL-13 had no impact on the HDAC5 expression, stimulation with the pro-inflammatory cytokine IFNγ did profoundly suppress HDAC5 mRNA expression similar to LPS (Fig.2A). But different form LPS treatment, IFNγ-treatment did not lead to an upregulation of HDAC5 expression within the first 60 min. of stimulation, neither did stimulation with MCP-1 or IL-4 + IL-13 (Fig.2B).

These findings of an early HDAC5 regulation pattern in macrophages in the presence of a bacteria-derived pro-inflammatory stimulus led to the question of the functional impact.

### Functional impact of HDAC5 over-expression

**Figure 3:**
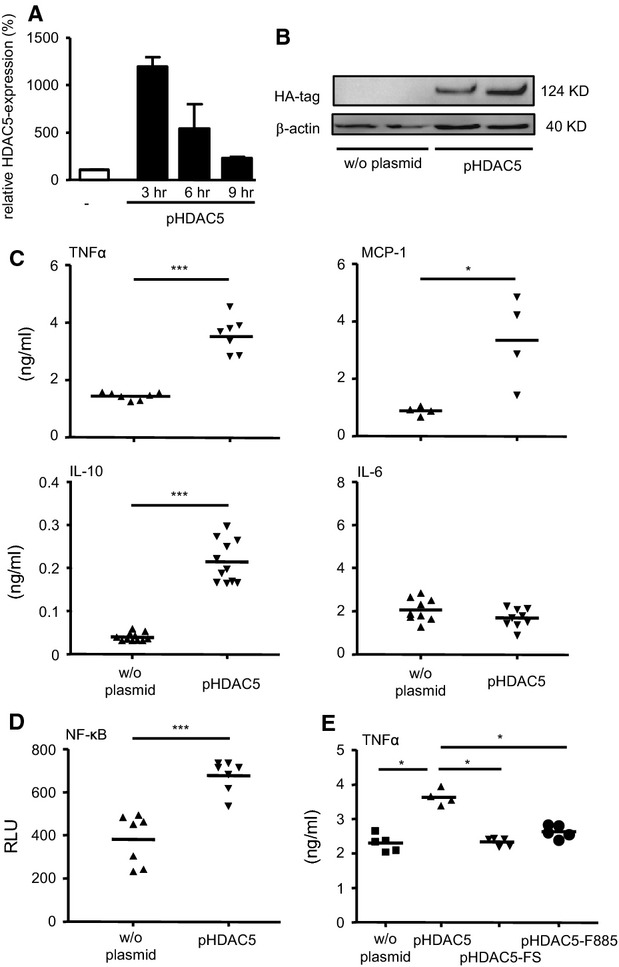
Regulatory impact of HDAC5 over-expression in RAW264.7 cells for cytokine response and NF-κB activation. (A) Cells were transfected with the HDAC5 expression plasmid pHDAC5 or remained non-transfected (w/o plasmid). Expression levels of HDAC5 mRNA were determined by qPCR relative to the untreated samples. (B) Lysates from transfected and non-transfected cells (10^5^) were analysed by Western blot for the HA-tag and β-actin as loading control. (C) Cells transfected with pHDAC5 were stimulated with LPS for 16 hrs before TNFα, MCP-1, IL-10 or IL-6 was determined from the supernatant. Median values and ranges of *n* = 2–8 from 2–4 independent experiments. (D) Cells transfected with NF-κB reporter plasmid in combination with pHDAC5 as indicated were incubated for 1 hr in the absence or presence of LPS. NF-κB activation was measured in relative light units. (E) Cells co-transfected with pHDAC5 and the plasmids coding for a frame shift mutant (pHDAC5-FS) or a mutant bearing an amino acid exchange (pHDAC5-F885) were stimulated with LPS for 16 hrs and production of TNFα was measured in the supernatant. (C–E) Median values and ranges of *n* = 4–11 from 2–4 independent experiments. *P < 0.05, ***P < 0.001 by Kruskal–Wallis test and Mann–Whitney test as post-test (A, E) or by Mann–Whitney test (C, D).

Over-expression of HDAC5 was established in RAW264.7 cells by transiently transfecting murine full-length HDAC5 under the control of a strong promoter. The kinetics as assessed at transcriptional level showed significantly more than 10-fold elevated levels of HDAC5 mRNA after 3 hrs that constantly declined within 9 hrs (Fig.3A) and returned to starting level within 24 hrs (data not shown). Independent from the presence of specific mRNA, high levels of HDAC5 protein were detected for at least 24 hrs (Fig.3B). Having established the cell model, the effect of HDAC5 expression on LPS induced cytokine secretion was examined (Fig.3C). Over-expressed HDAC5 resulted in a significant increase of TNFα, MCP-1 as well as IL-10 secretion, whereas the expression of IL-6 remained unchanged. There was also no change in the expression of the costimulatory molecules and activation markers CD80 and CD86 (data not shown). Excluding HDAC5-independent effects caused by the plasmid itself, a frame shift mutant disabling HDAC5 expression did not change the expression levels of TNFα (Fig.3D). The same result was achieved by replacing the histidine residue in position 885 for phenylalanine in the transiently over-expressed HDAC5 leading to a functional deficiency of the protein. Addressing the signalling pathways recruited for TNFα production, pHDAD5 was co-transfected with a NF-κB reporter plasmid into RAW264.7 cells (Fig.3E). Suggesting that HDAC5 might be directly involved in regulating the expression of this cytokine, a profound, about twofold increase in the activation of NF-κB could be detected in parallel to the HDAC5 over-expression.

### Functional impact of HDAC5 knock down

**Figure 4:**
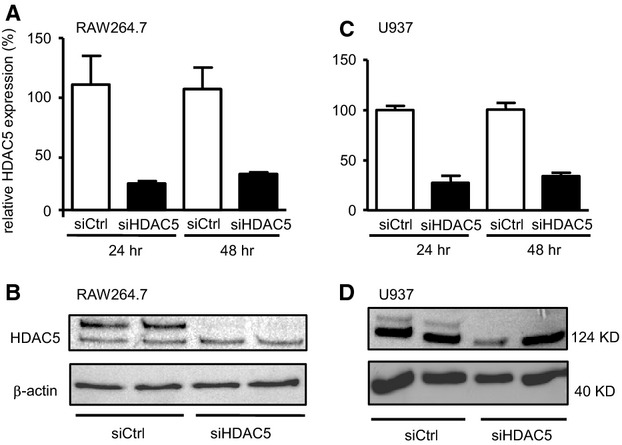
HDAC5 knock down *via* siRNA. Cells were transfected with siRNA targeting HDAC5 (siHDAC5) or with control siRNA (siCtrl). Expression levels of HDAC5 mRNA in RAW264.7 (A) or U937 cells (C) were determined by qPCR relative to the samples transfected with control siRNA at 24 and 48 hrs after transfection. Mean values ± SEM of *n* = 2–3. Lysates from transfected 10^5^ RAW264.7 (B) or 10^5^ U937 cells (D) 24 hrs after transfection were analysed by Western blot for HDAC5 and β-actin as loading control.

**Figure 5:**
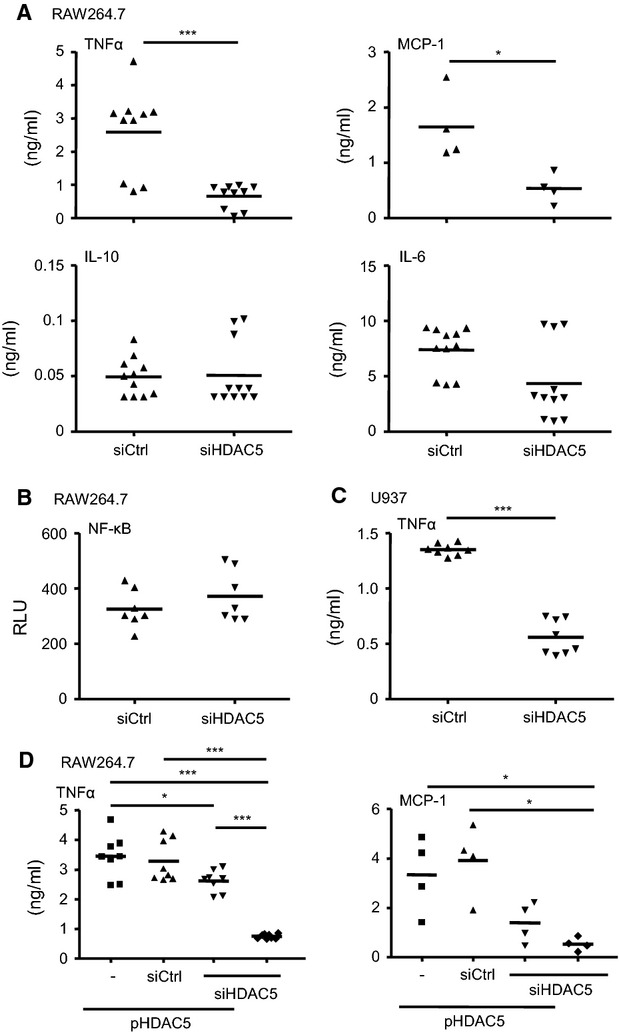
Effect of HDAC5 knock down on cytokine production and NF-κB activation. (A) RAW264.7 cells transfected with siRNA against HDAC5 (siHDAC5) or control siRNA (siCtrl) were stimulated with LPS for 16 hrs before concentrations of TNFα, MCP-1, IL-10 or IL-6 were determined from the supernatant. Median values and ranges of *n* = 4–10 from 3–4 independent experiments. (B) Cells transfected with NF-κB reporter plasmid in combination with siHDAC5 or siCtrl as indicated were incubated for 1 hr in the absence or presence of LPS. NF-κB activation was measured in relative light units. Median values and ranges of *n* = 7 from 2 independent experiments. (C) U937 cells transfected with siHDAC5 or siCtrl were treated with LPS for 16 hrs before production of TNFα was measured from the supernatant. Median values and ranges of *n* = 8 from 4 independent experiments. (D) RAW264.7 cells co-transfected with siHDAC5 or siCtrl and pHDAC5 were stimulated with LPS for 16 hrs before TNFα or MCP-1 in the supernatant was determined. Median values and ranges of *n* = 8 from 3 independent experiments. *P < 0.05, ***P < 0.001 by Mann–Whitney test (A, C) or by Kruskal–Wallis test and Mann–Whitney test as post-test (D).

The next step addressed the functional impact of HDAC5 by knocking down the enzyme using RNA interference. Transfection of RAW264.7 cells with the HDAC5-specific siRNA resulted in a profound suppression of HDAC5 mRNA which remained stable for at least 48 hrs (Fig.4A). The HDAC protein also was not found 48 hrs after transfection of the specific siRNA but was high in the control cells (Fig.4B). Comparable expression pattern were found for HDAC5 mRNA (Fig.4C) and protein (Fig.4D) if the human monocyte cell line U937 was also transfected with specific siRNA. As for the functional impact of the successful knock down, the cytokine and chemokine expression in response to LPS stimulation of transfected RAW264.7 and U937 cells was studied. Confirming the findings from the transient over-expression of HDAC5 described above, LPS stimulation in the HDAC5 knock down cells was followed by a robust suppression of TNFα and MCP-1, whereas IL-10 and IL-6 remained unchanged (Fig.5A). Again no change in the expression of CD80 or CD86 could be detected (data not shown). The knockdown of HDAC5 had no impact on NF-κB activity as shown by co-transfection of the siRNAs with the NF-κB reporter plasmid (Fig.5B). The profound reduction in TNFα expression in murine cells was confirmed with the human cells, when siHDAC5-treated U937 cells were analysed for the expression of this cytokine (Fig.5C).

To verify the specificity of the HDAC5 effect on LPS-induced cytokine expression RAW264.7 cells were co-transfected with the plasmid for HDAC5 over-expression and the HDAC5-specific siRNA. Tendencies of lowered expression of pro-inflammatory TNFα as well as of MCP-1 indicated a specific down-regulating effect of the HDAC5 specific siRNA compared to the control siRNA, even upon plasmid-driven high expression of HDAC5 (Fig.5D).

Effects regarding the production of TNFα and MCP-1 after knockdown of HDAC5 that were consistent with findings from the over-expression experiments emphasized the conclusion that HDAC5 regulates the pro-inflammatory capacity of macrophages.

## Discussion

Anti-inflammatory effects of general HDAC inhibition has been proven in various *in vitro* and *in vivo* models but the role of single HDAC in defined cell populations remains to be defined. Focusing on the role of HDAC5 in macrophages, we showed that HDAC5 expression was down-regulated by pro-inflammatory LPS or IFNγ. HDAC5 over-expression was associated with a significant increase of TNFα and MCP-1 paralleled by an activation of the NF-κB pathway. Supporting the concept of direct involvement of HDAC5 in the pro-inflammatory capacity of macrophages, HDAC5 knockdown by siRNA resulted in the suppression of cytokine production.

Establishing an impact of HDAC5 in innate immune cells and cell lines as suitable models, HDAC5, 7 and 9 are up-regulated during U937 differentiation into macrophages 25. Down-regulation of pro-inflammatory cytokine production by extracts of edible blue-green algae with generally decreased HDAC expression including HDAC5 and parallel inhibition of NF-κB activity in RAW264.7 cells and in murine bone marrow-derived macrophages link the anti-inflammatory effect to this central pathway 26.

The time-dependent decrease of HDAC5 mRNA after onset of LPS stimulation was concordant to earlier studies 26–28, but this immediate effect ceased within 24 hrs. Additionally to LPS, only IFNγ or CpG oligonucleotides affected the HDAC5 mRNA levels, which connects this modulation of HDAC5 expression to defined pro-inflammatory conditions and to the cross-talk between IFNγ signalling and the Toll-like receptor pathway, activated by LPS *via* TLR4 or CpG *via* TLR9 29. IFNγ sensitizes macrophages for TLR ligands 30,31 and STAT1 activity, induced by TLR4 and TLR9 signalling, mediates IFNγ-dependent gene regulation 32,33. These processes are accompanied by chromatin remodelling, where HDAC act as negative feedback regulators for LPS-inducible genes like Cox2 28. HDAC are also required for signalling via STAT1, where the activity is regulated by protein acetylation 35,36. We suggest that in macrophages especially HDAC5 is crucial to control inflammation caused by bacterial pathogen-associated molecular pattern including LPS and CpG or bypassed by IFNγ. Although TNFα is involved in intracellular localization and activity of HDAC *e.g*. by regulating shuttling of HDAC3 and HDAC4 into the nucleus 36 and diminishing HDAC1 activity by increased protein turnover 37, TNFα did not represent a potential autocrine feedback mechanism for HDAC5 to LPS stimulation. This held also true for other cytokines released by macrophages or known for affecting macrophages like TGFβ, IL-6 or MCP-1.

Enhanced induced NO synthase production upon HDAC5 over-expression in murine RAW264.7 cells or a reduced p65 activation underline the involvement in the defense of pathogens by macrophages but so far does not fully define the possible role for HDAC5 38,39. Knockdown of HDAC5 in alveolar epithelial cells, astrocytes and preadipocytes revealed no relation to inflammatory responses of the respective cell types 40–42. In our hands, transient over-expression of full-length HDAC5 in macrophages was associated with significantly augmented levels of pro-inflammatory TNFα and MCP-1 as well as of anti-inflammatory IL-10, while IL-6 levels remained unaffected. This was strengthened by the finding that knockdown of HDAC5 significantly lowered TNFα and MCP-1 levels. Assigning this functional impact to HDAC5, inactivating frame-shift mutation had no pro-inflammatory effect. Since a point mutation in the HDAC5 domain crucial for the formation of matrix-associated deacetylase bodies that are necessary in transcriptional regulation 23 also did not led to increased TNFα and MCP-1 secretion, we conclude that the integrity of this HDAC domain is indeed mandatory for the immune regulatory function of HDAC5 in macrophages. While in recent years, macrophages have been classified as M1 (pro-inflammatory) and M2 (anti-inflammatory) phenotypes, we do not propose a role of HDAC5 in macrophage polarization. In our system, not only ‘M1 cytokines’ like TNF and MCP-1 are affected by HDAC5 expression, but also IL-10 as classical anti-inflammatory cytokine.

HDAC inhibitors provide evidence that all HDAC can affect the NF-κB signalling recruited in the regulation of immune responses of macrophages 12,43. HDAC activity is also known as prerequisite to activate DNA polymerase II by NF-κB 44. Studying dependency of NF-κB activity from HDAC5 levels in an approved NF-κB reporter system, confirmed NF-κB signalling to be involved in the modulating activity of HDAC5 in macrophages.

Our study reveals a direct regulatory function of HDAC5 in the inflammatory response of macrophages. TLR4 or TLR9 stimulation induced immediate up- followed by fast down-regulation of HDAC5 expression. Defining HDAC5 expression levels by over-expression or by knockdown resulted in a concordant change mainly in the pro-inflammatory capacity of the macrophages with HDAC5 to be considered as part of the NF-κB activation process in a TLR-mediated pro-inflammatory response. Furthermore, it seems that the HDAC5 expression level at the time of activation (or shortly after) defines the following cytokine response. Hence, the early up-regulation of HDAC5 (Fig.1E and G) might be responsible for the cytokine release, while the following down-regulation for several hours could represent a compensatory regulation. Although the exact feedback mechanism remains unclear, autocrine cytokine signalling is not likely to be involved.

Taken together, the present study sheds new light on the role of individual HDAC in the activation of immune cells and appoints HDAC5 as a potential therapeutic target in diseases with massive macrophage activation like modulated as in autoimmune diseases or inflammation-associated cancer.
